# Impaired Functional Homotopy and Topological Properties Within the Default Mode Network of Children With Generalized Tonic-Clonic Seizures: A Resting-State fMRI Study

**DOI:** 10.3389/fnins.2022.833837

**Published:** 2022-06-02

**Authors:** Yongxin Li, Bing Qin, Qian Chen, Jiaxu Chen

**Affiliations:** ^1^Formula-Pattern Research Center, School of Traditional Chinese Medicine, Jinan University, Guangzhou, China; ^2^Department of Neurosurgery, Epilepsy Center, The First Affiliated Hospital, Jinan University, Guangzhou, China; ^3^Department of Pediatric Neurosurgery, Shenzhen Children’s Hospital, Shenzhen, China

**Keywords:** generalized tonic-clonic seizure children, default-mode network, voxel-mirrored homotopic connectivity, graph theory, support vector machine

## Abstract

**Introduction:**

The aim of the present study was to examine interhemispheric functional connectivity (FC) and topological organization within the default-mode network (DMN) in children with generalized tonic-clonic seizures (GTCS).

**Methods:**

Resting-state functional MRI was collected in 24 children with GTCS and 34 age-matched typically developing children (TDC). Between-group differences in interhemispheric FC were examined by an automated voxel-mirrored homotopic connectivity (VMHC) method. The topological properties within the DMN were also analyzed using graph theoretical approaches. Consistent results were detected and the VMHC values were extracted as features in machine learning for subject classification.

**Results:**

Children with GTCS showed a significant decrease in VMHC in the DMN, including the hippocampal formation (HF), lateral temporal cortex (LTC), and angular and middle frontal gyrus. Although the patients exhibited efficient small-world properties of the DMN similar to the TDC, significant changes in regional topological organization were found in the patients, involving the areas of the bilateral temporal parietal junction, bilateral LTC, left temporal pole, and HF. Within the DMN, disrupted interhemispheric FC was found between the bilateral HF and LTC, which was consistent with the VMHC results. The VMHC values in bilateral HF and LTC were significantly correlated with clinical information in patients. Support vector machine analysis using average VMHC information in the bilateral HF and LTC as features achieved a correct classification rate of 89.34% for the classification.

**Conclusion:**

These results indicate that decreased homotopic coordination in the DMN can be used as an effective biomarker to reflect seizure effects and to distinguish children with GTCSs from TDC.

## Introduction

Epilepsy is one of the most common serious neurological brain disorders, affecting over 65 million people worldwide (Thurman et al., 2011). Patients with epilepsy are clinically characterized by seizure symptoms and impaired consciousness. Generalized tonic-clonic seizures (GTCS) encompass a group of seizure types that are characterized by generalized spike-wave discharges (2.5–5 Hz), involving the bilateral hemispheres during seizures (Ji et al., 2014). Using routine MRI, focal anatomical brain lesions usually cannot be detected in patients with GTCS. The clinical characteristics of patients with GTCS include muscle rigidity, violent muscle contraction of the entire body, and complete loss of consciousness (Zhang et al., 2011). Pieces of evidence have shown significant cognitive and psychosocial impacts in patients with GTCS, including impaired attention, memory, and executive function (Hommet et al., 2006; Paige and Cavanna, 2013). For people with GTCS, their quality of life is affected. As a result, this type of epilepsy is attracting much medical attention in clinical practice (Kenyon et al., 2014; Ryvlin and Beniczky, 2020). Although many effects have been observed in the past, the neural mechanisms underlying the GTCS remain unclear.

Previous studies on epilepsy mainly used information on clinical manifestations and EEG data (Myers et al., 2018; Trinka et al., 2019). The accepted view is that epileptic seizures are induced by an imbalance between excitatory and inhibitory activities (Fisher et al., 2017a). Normal neuronal processing was disrupted in patients with epilepsy. Recently, neuroimaging techniques have been applied to explore the neuromechanism of the human brain, and epilepsy is considered as a functional brain network disorder by this technique (Engel et al., 2013; Fitsiori et al., 2019; Jia et al., 2020; Goodman and Szaflarski, 2021). For GTCS, this type of epilepsy belongs to generalized seizures, which are widespread throughout the entire brain or bilaterally distributed networks (Fisher et al., 2017a). This bilateral epilepsy feature can lead us to think about the brain organization by the fMRI method. Growing evidence in the neuroimaging domain has found that patients with GTCS showed bilateral impairments of their functional network, which is thought to be associated with the abnormalization of multiple interconnected brain systems (Wang et al., 2011; Jia et al., 2020; Li et al., 2020a; Parsons et al., 2020). The alterations in brain activity and connections of the patients with GTCS were involved in some brain systems, including the default mode network (DMN), thalamocortical system, and visual and dorsal attention networks (Wang et al., 2011, 2019a; Liu et al., 2017). Regarding the DMN, previous studies have detected that this network directly contributes to internal mentation (Buckner et al., 2008). Patients with GTCS showed a significant decrease in functional connectivity (FC) and spontaneous activity in the DMN (Song et al., 2011; Parsons et al., 2020). The alterations in brain activity in the DMN were mainly located in the medial prefrontal cortex, posterior midbrain regions, and lateral parietal cortex for epileptic patients with GTCS (Hamandi et al., 2006; Blumenfeld et al., 2009). The findings from these previous studies indicated that abnormal connectivity and activity in the DMN may be the neural substrate of the impaired consciousness and cognitive impairments in patients with GTCS. Although these previous studies have found changes in the DMN in GTCS, the specific role of the DMN in measuring seizure induced network organization, and detecting neuroimaging biomarkers still needs further investigation. Therefore, the ability to identify and track the specific DMN abnormalities in GTCS may provide an objective biomarker for GTCS diagnosis and understanding the neural mechanism of GTCS.

Combining the previous neuroimaging findings and clinical characteristics of GTCS, we can see that interhemispheric synchronization would be affected in patients with GTCS. During seizures, generalized spike-wave discharges may induce changes in interhemispheric communication. Thus, alterations in the functional interaction between the bilateral hemispheres have recently attracted people’s attention. One study used multimodal MRI data to characterize interhemispheric functional and anatomic connectivity in patients with GTCS (Ji et al., 2014). Compared with the healthy controls, patients with GTCS showed both increased and decreased interhemispheric functional connectivity. The length for the fiber bundles connecting the bilateral anterior cingulate cortex and the bilateral cuneus was short in patients. Meanwhile, a neuroimaging study based on the voxel-mirrored homotopic connectivity (VMHC) method was used to study the resting state functional connectivity between the two hemispheres (Yang et al., 2014). Patients with GTCS showed significant increases in VMHC in the anterior cingulate and medial prefrontal gyrus. No areas showed a significant decrease in VMHC in patients with GTCS. The VMHC in the bilateral thalamus, orbital frontal cortex, and cerebellum showed negative correlations with illness duration. We can see that some results of the above two studies are inconsistent. For example, the VMHC values in the inferior frontal gyrus of patients showed a significant decrease in one study, but not in the other. The possible reason for this inconsistency may be that one study only included adults, and the other study included both adults and children. However, in the above two studies, most regions showing significant changes were mainly located in the DMN, which may be critical to the pathophysiology of patients with GTCS. Considering the bilateral seizure feature of GTCS and the inconsistent results in these previous studies, researching the interhemispheric communication in the DMN of patients with GTCS would provide useful information to understand this disease. In the present study, we focused on the interhemispheric functional connectivity of the DMN to detect the specific role of this network in GTCS.

Additionally, the subjects of these previous studies were adult patients with GTCS. Only a few studies have focused on the brain organization and activity of children with GTCS. One recent neuroimaging study in patients with epilepsy with GTCS found that both children and adult patients showed increased functional diversity in frontocentral neocortical regions (Wang et al., 2019a). Another study from our group on children with GTCS also detected a significant reduction in gray matter volume and an increase in spontaneous activity in the temporal lobe, hippocampus, thalamus, and other deep nuclei in patients (Wang et al., 2018). The consistent results from these two studies were that significant changes of brain functional activity in the DMN were detected of children with GTCS. The graph theory method was also applied in children with GTCS from our group. Children with GTCS still exhibited efficient small-world properties of their whole brain gray matter structural covariance network and functional network similar to the normal controls (Li et al., 2020a,b). Significant changes in nodal betweenness of the structural network were located in the thalamus, temporal pole, and some regions of the DMN (Li et al., 2020b). The functional connections within the DMN were decreased significantly, and the internetwork connections were increased significantly in children with GTCS (Li et al., 2020a). Both graph theory studies in children with GTCS demonstrated a disrupted topological organization in some regions of the brain’s functional and structural network. Previous studies have consistently pointed out that connection abnormalities of the brain network still exist in children with GTCS. However, the functional organization and topological properties within the DMN are still unclear in children with GTCS. Considering the important role of the DMN in the human brain, researching the functional organization of the DMN in children with GTCS can enrich our knowledge to understand the pathophysiology of this type of epilepsy.

Thus, the aim of the present study was to explore the alteration of interhemispheric functional connectivity and topological properties within the DMN in children with GTCS. According to clinical and previous neuroimaging results, we hypothesized that children with GTCS would show abnormal functional connectivity between the left and right regions within the DMN. The specific change in interhemispheric connectivity can reflect the epilepsy’s clinical manifestations. To test this hypothesis, we combined the VMHC method and graph the theory method on resting-state fMRI data in children with GTCS. Using the VMHC method, functional homotopy between hemispheres can be quantified for children with GTCS. Graph theory can be used to quantify network topology and brain connections of the DMN in children with GTCS. We used these two methods from different perspectives to study the interhemispheric connectivity changes in the DMN. The consistent results from these two methods were selected as regions of interest for the correlation analysis with the clinical characteristics. The interhemispheric connectivity values of the regions of interest were also extracted as features for further machine learning to detect whether these functional homotopies in the DMN can correctly distinguish the children with GTCS from the typically developing children.

## Materials and Methods

### Subjects

Twenty-four children with GTCS (9 females; mean ages: 69.94 ± 46.36 months) were included in the study. All the patients were diagnosed with GTCS. The inclusion criteria for the patient group were as follows: (1) met the criteria of GTCS diagnosis according to the current International League Against Epilepsy seizure type classification (Fisher et al., 2017b), such as limb movement, loss of consciousness during seizures, and no partial seizures; (2) a specific pattern of electrophysiological activity on electroencephalogram (generalized spike-and-wave or poly-spike-wave discharges); and (3) no abnormality was detected for all the patients in routine MRI examinations. Each patient was treated with at least one antiepileptic drug (AED: topiramate, valproic acid, oxcarbazepine, and/or levetiracetam, 11 patients with 1 AED, 10 patients with 2 AEDs, and 3 patients with 3 AEDs) to control seizures before imaging data collection. All the patients were seizure free for at least 2 days prior to MRI examination. A group of typically developing children (TDC, 10 females, 24 males, mean age: 69.38 ± 28.82 months) were included for comparisons with patient cohorts. All TDC had no history of neurological disorders or psychiatric illnesses. During the MRI scanning, the participants under the age of four were sedated with 10% chloral hydrate to reduce their head movement (dosage: 50 mg/kg, the maximum dose is 1 g). Eighteen participants (9 children with GTCS and 9 TDC) of this study were under 4 years. Demographic and clinical information of both groups can be found in Table 1.

**TABLE 1:** Demographic and clinical information data of the subjects.

Characteristics	Patient group		Control group		Comparisons
	(Mean ± *SD*)	(median + IQR)	(Mean ± *SD*)	(median + IQR)	
Gender (female/male)	9/15	\	10/24	\	*X*^2^ = 0.42 (*P* = 0.52)
Age (month)	69.94 ± 46.36	55 + 65	69.38 ± 28.82	58 + 47	*t* = 0.06 (*P* = 0.95)
Seizure onset age (month)	37.35 ± 46.22	18 + 30	\	\	\
Duration (month)	32.58 ± 31.20	27 + 41	\	\	\

*SD, standard deviation; IQR, inter-quartile range.*

Before the image data were collected, the study purpose, procedures, possible risks, and discomforts were explained to the participants and their parents or the guardians. The parents or the guardians of all the participants gave written informed consent. This study was approved by the Ethical Committee of the Shenzhen Children’s Hospital.

### Data Acquisition

Data were acquired using a German Siemens Trio Tim 3.0T scanner (MAGNETOM, Germany, 8-channel head coil) at the Shenzhen Children’s Hospital, Shenzhen, China. Foam cushions and earplugs were used for all the subjects to reduce head movements and machine noise, respectively. High-resolution three-dimensional (3D)T1-weighted MPRAGE images covering the entire brain were acquired for all the subjects: TR (repetition time, ms) = 2,300; TE (echo time, ms) = 2.26; FOV (field of view, mm) = 200 × 256; acquisition matrix = 200 × 256; 160 sagittal slices; slice thickness (mm) = 1; flip angle (degree) = 8. Resting-state fMRI data were collected axially by using an echo-planar imaging sequence with 130 volumes: TR (ms) = 2,000, TE (ms) = 30, FOV (mm) = 220 × 220, matrix size = 94 × 94, slice thickness (mm) = 3, flip angle (degree) = 90, 36 interleaved axial slices covered the entire brain. During the imaging scanning, the participants over the age of four were instructed to keep still with their eyes closed, remain awake, and instructed not to think about anything. All the participants were lying quietly, as motionless as possible. To avoid falling asleep of these participants, we observed throughout the whole scanning process, and asked their conditions after that. During the scanning, we observed throughout the whole scanning process. The T1 data were checked during the scanning process. During the scanning process, T1 images were scanned first and then scanned the resting-state image. If there are serious head-moving artifacts, we would tell the participant to keep still and rescan the T1 image. If the second scan still existed serious head-moving artifacts, we would stop the data collection of this participant and not include in the data analysis. During the whole data collection process, 12 subjects (5 TDCs and 7 patients) failed to obtain complete imaging connection by the above reason.

### Data Preprocessing

The resting-state fMRI data were preprocessed using the data assistant software DPABI (Yan et al., 2016), which runs on MATLAB 8.2 (Mathworks, Natick, MA, United States). The first 10 time points were removed to ensure magnetization equilibrium. The remaining volumes were corrected by the acquisition time delay among different slices and realigned to the first volume to correct the head motions. All the participants had less than 3-mm maximum displacement in the *x*-, *y*-, or z-axis and 3° of angular motion during data acquisition. Subsequently, each subject’s high-resolution anatomical image was co-registered to the mean functional images by rigid body transformation. The transformed structural images were then segmented in to gray matter, white matter, cerebrospinal fluid by using a unified segmentation algorithm, and normalized to the Montreal Neurological Institute (MNI) space by using a 12-parameter non-linear transformation. The transformation parameters were applied to the functional images and resampled the functional images to a 3-mm isotropic voxel. The functional images were spatially smoothed with a Gaussian kernel of 6-mm full width at half maximum. The smoothed images were masked by the default gray matter mask of the software. Then, the masked images were subjected to temporal bandpass filtering (0.01–0.08 Hz) and linear detrending to reduce the effect of low-frequency drifts and physiological high-frequency noise.

To minimize the potential effects of head motion on subsequent graph theory analyses, mean framewise displacement values were also calculated during the realignment steps and compared between the two groups. For each subject, the mean framewise displacement values were the across translational and rotational directions of scan-to-scan deviations between two images (Power et al., 2012). No participant was excluded, and the mean framewise displacement value exceeded 0.5 mm. The framewise displacement was not significantly different between the two groups (the TDC: mean = 0.12 ± 0.10 mm; the children with GTCS: mean = 0.13 ± 0.09 mm). Spurious covariates and their temporal derivatives, including Friston-24 head motion parameters, white matter signals, and cerebrospinal fluid signals, were removed from the data using linear regression.

### Interhemispheric Correlation and Statistical Analysis

The VMHC was also calculated with the data-assistant DPABI. VMHC assumes symmetric morphology between hemispheres. To account for differences in the geometric configuration of the cerebral hemispheres, we firstly averaged the normalized T1 images of all the subjects to create a mean normalized T1 image. This mean T1 image was then averaged with its left-right mirrored version to generate a group-specific symmetrical T1 template. Then, the individual T1 images in MNI space were non-linearly registered to the symmetrical T1 template, and those transformations were applied to the above-processed functional data. For each subject, the homotopic connectivity was computed as the Pearson’s correlation coefficient between the residual time series of each voxel and that of its symmetrical interhemispheric counterpart (Zuo et al., 2010). Correlation values were then Fisher *z*-transformed to improve the normality. The resultant values were referred to as the VMHC and were applied for the group comparisons.

To test for regional group differences in VMHC, individual-level VMHC maps were entered into a group-level voxel wise *t*-test. Significant differences of VMHC between the children with GTCS and the TDC were set at the threshold of voxel wise *p* < 0.01 (FDR corrected) and cluster size of 6.

### Topological Analysis in Default-Mode Network and Statistical Analysis

Because the present study mainly focused on the functional abnormality in DMN, we also used graph theory to investigate the alterations of brain topology in DMN. The brain network topological characterizations were analyzed using GRETNA^1^ (Wang et al., 2015). First, we defined 17 anatomic nodes to examine brain topology in DMN (Supplementary Table 1). Each node was defined as an 8-mm radius sphere centered on the MNI coordinates from a previous study (Andrews-Hanna et al., 2010). These nodes were grouped into two hubs, including posterior cingulate cortex (PCC) and anterior medial prefrontal cortex (aMPFC), respectively, and two subsystems, a “dorsal medial prefrontal cortex” (dMPFC) subsystem and a “medial temporal lobe” (MTL) subsystem. Second, the averaged time series from each node were extracted and then computed the temporal correlation among time series using Pearson correlation. Third, all resulting correlation coefficients were transformed into *z*-scores using Fisher’s *z*-transformation to improve the normality of the correlation coefficients. The normalized correlation value of each pair was regarded as the network edges. Thereafter, a 17- × -17 correlation matrix was produced for each subject. A binary matrix was obtained according to a predefined threshold (see below for the “Threshold” selection), where edges with positive correlation values were set to 1 and, otherwise, set to 0. Finally, different levels of network topological properties were performed, including global network metrics and regional nodal properties. The global network architecture of the functional networks was characterized using small-world [small-worldness (σ), clustering coefficients (*C*_p_), normalized clustering coefficient (γ), characteristic path length (*L*_p_), normalized characteristic path length (λ)], and network efficiency [local efficiency (*E*_loc_) and global efficiency (*E*_g_)]. The regional characteristics, such as nodal efficiency, nodal local efficiency, nodal shortest path, nodal degree centrality, and betweenness centrality, were also assessed. The definitions of these network properties can be found in previous graph studies (Bassett and Bullmore, 2006; Sporns and Honey, 2006).

In the present study, a threshold of connection sparsity, S, was used for all functional matrices. Currently, there is no criterion for selecting a single threshold for constructing functional brain networks. Instead of selecting a single threshold, we investigated the topological properties of each matrix over a wide range of threshold levels (0.1–0.5 with an interval of 0.01). Using this approach, all resulting networks have the same number of edges. The range of sparsity values was chosen here to allow small-world network properties to be properly estimated. At the lower bound of the range, the networks of both groups were not fragmented. For densities above 0.45, the graphs became increasingly random (σ < 1.5) (Stam and Reijneveld, 2007). This reference found that the small-world model synchronized as rapidly as a fully random network for thresholds > 0.5. So, the maximum threshold was selected at 0.5 in the present study to ensure that the thresholded network displayed small-worldness.

We also calculated the area under the curve (AUC) for each network metric (global and local topological properties). The AUC is a summarized scalar, which can reflect the topological characterization of brain networks for each network metric. AUC was independent of single threshold selection and sensitive to topological alterations in brain disorders (Suo et al., 2015). Between-group differences in the AUCs were analyzed using independent sample *t*-test with age and gender as covariates (*p* < 0.05). Between-group differences were identified in the nodal metrics.

We used a network-based statistic (NBS) approach for the functional connectivity networks to localize the specific connected components, which reflects the functional connections in DMN that differed between each pair of groups (Zalesky et al., 2010). A set of suprathreshold links among all connected components was defined using the NBS method. The non-parametric permutation method was used to estimate the significance for each component (1,000 permutations). The threshold (*p* < 0.05) was adopted to address the comparisons in functional connectivity. Using the above process, significant between-group differences in the network metrics in the DMN were identified.

### Brain-Behavioral Relationships

We further calculated the brain-behavioral relationships in the patient group. The statistical analysis results of VMHC and functional connectivity in the DMN were combined. The consistent results in both analyses were selected as our region-of-interest (ROI). The mean VMHC values and the mean FC values were extracted in these ROIs, respectively. The correlations between these image indexes and the clinical characteristics (epilepsy duration and onset age of the first seizure) were calculated. And, also, the correlation between the mean VMHC values and the mean FC values in the ROIs was also calculated. The threshold (*p* < 0.05) was adopted to address the correlation results. During the comparison process, age and sex were controlled.

### Support Vector Machine Analysis

The support vector machine (SVM) method was operated using PRoNTo (Pattern Recognition for Neuroimaging Toolbox) software version 2.0^2^ in MATLAB (Abbasi and Goldenholz, 2019; Wissel et al., 2020). This method was applied to test the ability to differentiate children with GTCS from the TDC using the extracted VMHC values in abnormal brain regions. The regions showed significant difference between groups both in VMHC results, and FC results were selected and the signals of VMHC values in these regions were used as features of the SVM classifier. A “leave-one-out” method was used during the cross-validation step (Li et al., 2014). The statistical significance of the observed classification accuracy was estimated by permutation tests (Zeng et al., 2012). This involved repeating the classification procedure 1,000 times. The number of permutations achieving higher sensitivity and specificity than the true labels was used to derive a *p*-value. Statistical significance of classification accuracy was determined by this process.

## Results

### Group Differences in Voxel-Mirrored Homotopic Connectivity

**FIGURE 1:**
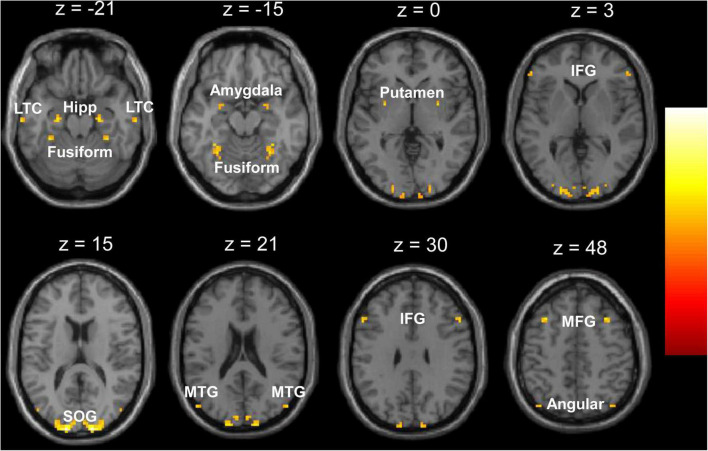
Regions showing significant decrease of voxel-mirrored homotopic connectivity (VMHC) in the children with generalized tonic-clonic seizures (GTCS). The threshold of resulting statistical maps was a combination of *p* < 0.01 (FDR correction) and minimum cluster number of 6. Hipp, hippocampus; LTC, lateral temporal cortex; MTG, middle temporal gyrus; SOG, superior occipital gyrus; IFG, inferior frontal gyrus; MFG, middle frontal gyrus.

There were no significant differences in age or gender distribution between the two groups (see Table 1). Compared with the TDC, the children with GTCS showed a significant decrease in VMHC in some regions, such as the superior occipital gyrus, hippocampal formation (HF), fusiform, putamen, amygdala, angular, lateral temporal cortex (LTC), middle temporal gyrus, inferior and middle frontal gyrus (see Figure 1). No areas showed a significant increase in VMHC in the patient group. Table 2 illustrates the details of group differences in VMHC.

**TABLE 2:** Significant group differences in voxel-mirrored homotopic connectivity (VMHC).

Cluster location	Statistical values		Peak (MNI)		
	Cluster size	*t*-value	*x-*	*y-*	*z-*
Control > patient					
Superior occipital gyrus	149	8.11	±15	−99	15
Middle frontal gyrus	14	5.36	±33	21	48
Hippocampus	10	5.23	±24	−12	−21
Lateral temporal cortex	6	5.23	±66	−20	−21
Fusiform	20	5.22	±30	−54	−15
Putamen	7	4.95	±30	0	0
Inferior frontal gyrus	6	4.84	±54	21	30
Middle temporal gyrus	6	4.80	±51	−78	21
Angular	6	4.68	±39	−75	45
Amygdala	6	4.57	±27	0	−12
Patient > control	No				

*The MNI coordinates and t-values for the local maxima of the centers of the voxel clusters. The threshold for significant clusters reported here was set at p < 0.01 (FDR corrected) and cluster size of 6.*

*VMHC, voxel-mirrored homotopic connectivity; MNI, Montreal Neurological Institute.*

### Group Differences in Global Network Metrics in Default-Mode Network

**FIGURE 2:**
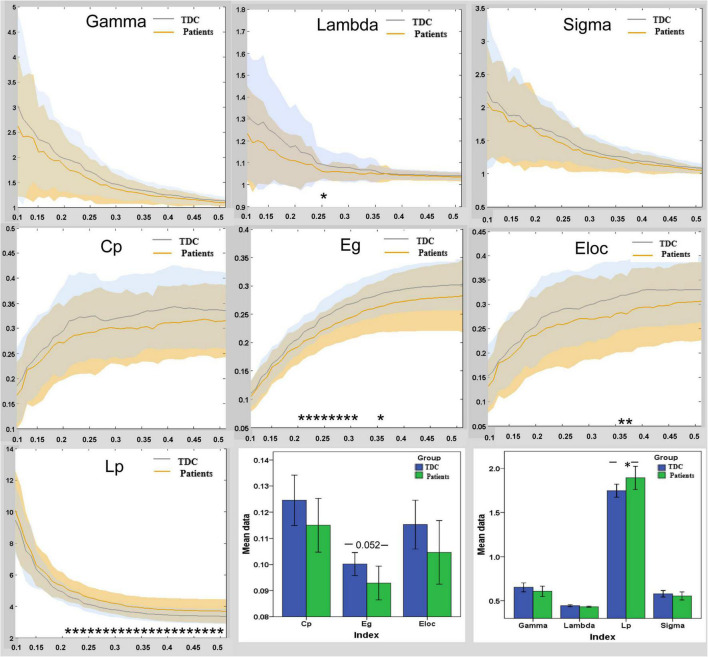
Group differences in global functional properties of brain topology in DMN between the patient and TDC. In the range of sparsity (0.1 ∼ 0.5), the functional networks of DMN in both groups exhibited a small-world property. Bar plots of the global efficiency, local efficiency, clustering co-efficiency, gamma, lambda, *L*_p_, and sigma for the children with GTCS and TDC. Asterisks (*) indicate a significant difference between the two groups. *E*_g_, global efficiency; *E*_loc_, local efficiency; *C*_p_, clustering coefficient; *L*_p_, characteristic path length; TDC, typically developing children.

Children with GTCS and TDC showed small-world organization (σ > 1) of the brain functional connectome in the DMN: high normalized C_p_ (γ > 1) and similar normalized Lp (λ ≈ 1) (Figure 2). The result was unified using a metric called small-worldness (σ > 1). Compared to the TDC, the patients showed significantly increased *L*_p_ values (*t* = 2.13, *p* = 0.038) in the functional network of the DMN. No significant differences in other global network parameters were observed (Figure 2).

### Group Differences in Regional Topological Organization in Default-Mode Network

Brain regions in the DMN with significant intergroup differences were identified (*p* < 0.05, Table 3). The children with GTCS showed decreased Cp in the right temporal parietal junction (TPJ). Decreased nodal efficiency was found in the left TPJ, bilateral LTC, and left HF. For the patient group, significant decreases in nodal local efficiency were found in the right TPJ and LTC. Near significant decreases in nodal degree centrality were found in the left LTC and left HF. Significant increases in nodal betweenness centrality were found in the left temporal pole (TempP) of the children with GTCS.

**TABLE 3:** Default-mode network (DMN) regions showing disrupted nodal topologic properties in children with GTCS compared with the controls.

Regions	Cluster coefficient (*t*/*p*)	Nodal efficiency (*t*/*p*)	Nodal local efficiency (*t*/*p*)	Degree centrality (*t*/*p*)	Betweenness centrality (*t*/*p*)
L TPJ		2.29/0.026		–	–
R TPJ	**2.08/0.042**	–	**2.08/0.042**	–	–
L LTC	1.89/0.065	**2.04/0.049**		2.0/0.051	–
R LTC	–	**2.39/0.02**	**2.11/0.039**	–	–
L TempP	–	–	–	–	**−2.49/0.016**
L HF	–	**2.40/0.02**	–	1.99/0.051	–

*R, right hemisphere; L, left hemisphere; TPJ, temporal parietal junction; LTC, lateral temporal cortex; TempP, temporal pole; HF, hippocampal formation. Significant correlation results were marked in bold.*

### Group Differences in Functional Connectivity

**FIGURE 3:**
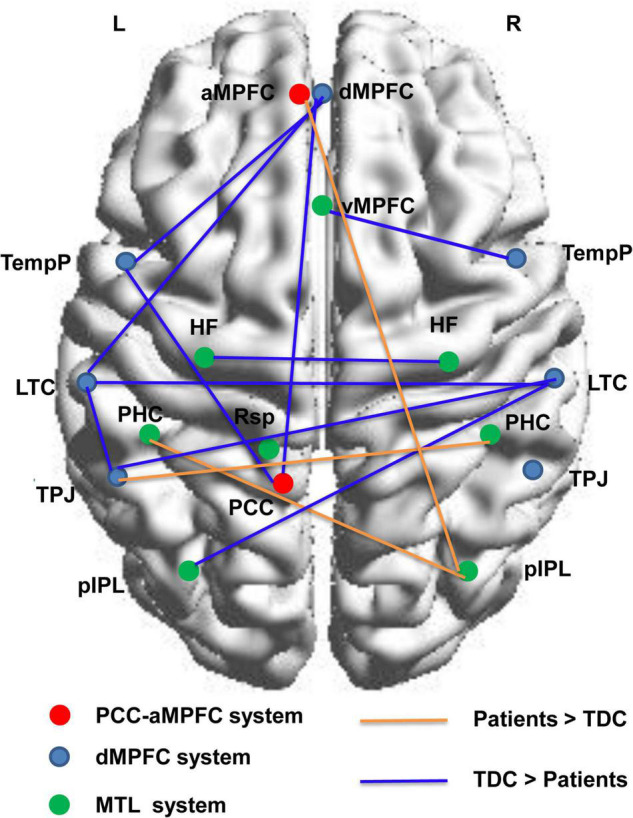
Connected networks in the DMN that showed significant changes of functional connections in the children with GTCS. The orange lines indicate the FC values are increased in the patient group, and the blue lines indicate the FC values are decreased in the patient group. The threshold is set at *p* < 0.05. L, left hemisphere; R, right hemisphere; TDC, typically developing children.

Based on NBS analysis, a decreased functional connectivity network in the DMN with 11 nodes and 10 connections was identified in the patient group, involving the bilateral LTC, bilateral HF, dMPFC, left TempP, left PCC, left TPJ, and left posterior inferior parietal lobule (pIPL). In particular, the functional connectivity between the bilateral HF and between the bilateral LTC showed a significant decrease in the patient group. An increased functional connectivity network with 5 nodes and 3 connections was identified in the patients, involving the bilateral parahippocampal cortex (PHC), left TPJ, right pIPL, and aMPFC. Node locations and significant changes in their connections are visualized in Figure 3.

### Relationship Between the Network Metrics and Epilepsy Duration

**FIGURE 4:**
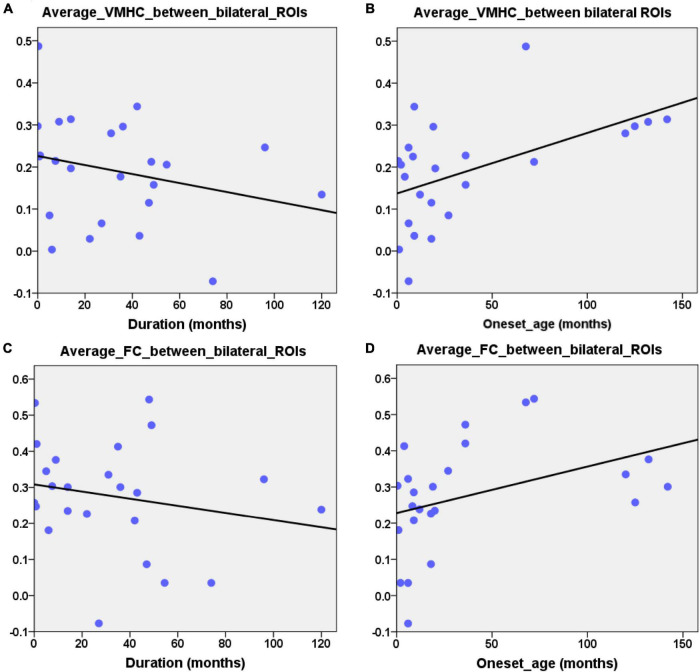
Partial correlation between the neuroimage indexes and the clinical characteristic. **(A)** Significantly negative correlation between epilepsy duration and the average VMHC values of ROIs (bilateral HF and LTC, *r* = –0.445, *p* = 0.038). **(B)** Significantly positive correlation between the first seizure onset age and the average VMHC values of ROIs (bilateral HF and LTC, *r* = 0.445, *p* = 0.038). **(C,D)** Scatterplot showing correlation between the average FC of bilateral ROIs (HF and LTC) and clinical characteristic (epilepsy duration: *r* = –0.281, *p* = 0.205, the onset age of first seizure: *r* = 0.281, *p* = 0.205). Age and gender were controlled during the above correlation analyses.

**FIGURE 5:**
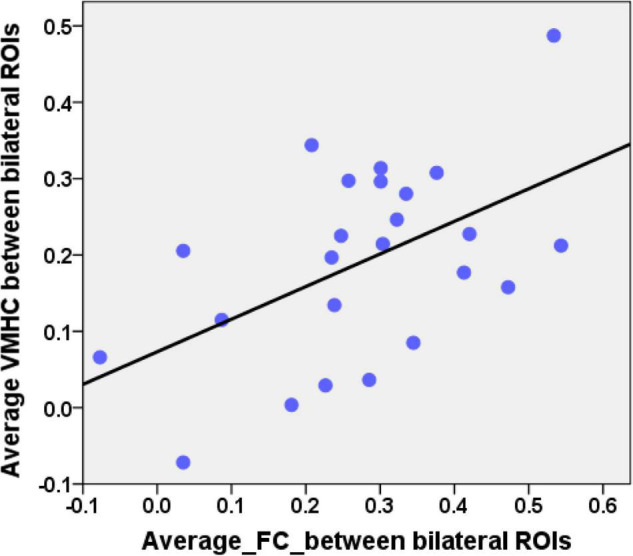
Correlation between the average VMHC values and the average FC values in patients. The average VMHC values were extracted from the bilateral HF and bilateral LTC of the group comparison results of the whole brain VMHC analyses. The average FC values were extracted from the bilateral HF and bilateral LTC of the NBS results of the DMN. Scatterplot showing significantly positive correlation between the average VMHC values and the average FC values in patients (*r* = 0.512, *p* = 0.015). Age and gender were controlled during this correlation analysis.

Combining the group comparison results of VMHC and NBS results of FC in the DMN, we found that the consistent results between the two methods mainly focused on the bilateral HF and bilateral LTC. Therefore, we selected these regions as our ROIs. The mean VMHC values and the FC values between the bilateral ROIs were extracted. Partial correlations between the epilepsy duration and the mean VMHC values of the bilateral ROIs (bilateral HF and bilateral LTC, *r* = −0.445, *p* = 0.038, Figure 4A) showed a significant correlation in the patient group. The mean VMHC values of the bilateral ROIs (bilateral HF and bilateral LTC) also showed significant correlations with the onset age of the first seizure (partial correlation: *r* = 0.445, *p* = 0.038, Figure 4B). The mean FC between the bilateral ROIs (bilateral HF and bilateral LTC) showed no significant correlation with the clinical characteristics (epilepsy duration, partial correlation: *r* = −0.281, *p* = 0.205 and onset age of the first seizure, partial correlation: *r* = 0.281, *p* = 0.205, Figures 4C,D). Additionally, after extracting the imaging index from the bilateral HF and bilateral LTC, the mean VMHC showed a significantly positive correlation with the mean FC (partial correlation: *r* = 0.512, *p* = 0.015, Figure 5). All correlation analyses were controlled for age and sex.

### Support Vector Machine Classification

**FIGURE 6:**
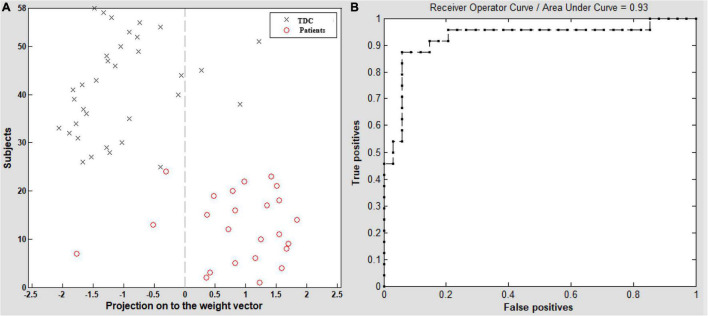
A classification plot **(A)** and a receiver operating characteristic (ROC) curve **(B)** for the comparison between groups. VMHC maps of 24 children with GTCS and 34 TDC were used for classification, which yielded an accuracy of 89.34% (87.50% sensitivity, 91.18% specificity), statistically significant at *p* < 0.001. TDC, typically developing children.

Based on the group comparison and the neuroimaging-clinical correlation results, we selected the bilateral HF and bilateral LTC as a mask. The VMHC in this mask was extracted as a feature in the model. Figure 6 shows the result of the SVM classification between 24 children with GTCS and 34 TDC based on the feature of VMHC in the bilateral HF and bilateral LTC derived from resting-state fMRI. The analysis of SVM classification achieved an accuracy of 89.34%, which was statistically significant at *p* < 0.001. The overall classification accuracy of the algorithm measures its ability to correctly classify an individual as a patient with GTCS or TDC. The model obtained a sensitivity of 87.50% and specificity of 91.18%. The area under the receiver operating characteristic curve (AUC) value was 0.93.

## Discussion

In the present study, we combined VMHC and graph-based theoretical approaches to investigate the functional alterations in the DMN of children with GTCS. The children with GTCS showed a significant decrease in VMHC in some regions, most of which belong to the DMN, such as the HF, LTC, and angular and middle frontal gyrus. Although the patients exhibited efficient small-world properties of their functional default mode network that were similar to the TDC, the DMN of the patients also exhibited a significant increase in *L*_p_. Group differences in regional topological organization were found in the areas of the bilateral TPJ, bilateral LTC, left TempP and left HF. In the DMN, the regions that exhibited a significant decrease in network connectivity were mainly within the dMPFC subsystem, and the regions that exhibited a significant increase in network connectivity were mainly between these subsystems in the children with GTCS. The FCs between the bilateral ROIs (left HF vs. right HF and left LTC vs. right LTC) were decreased significantly in patients, which was consistent with the VMHC results of the present study. The average VMHC values in bilateral HF and LTC were significantly correlated with the epilepsy duration and the onset age of first seizure in children with GTCS. The VMHC values in the bilateral HF and LTC also showed a significant correlation with the FC values among similar regions. Machine learning showed that the feature of VMHC values in the bilateral HF and bilateral LTC exhibited discriminative power in the classification of the children with GTCS from the TDC (accuracy of 89.34%). Together, our findings indicated that the brain functional homotopy, network architecture, and communities in the DMN of the children with GTCS were changed significantly. Our results provided preliminary evidence that the functional homotopy and topological organization of the DMN were disrupted in children with GTCS. The significant correlation results and the discriminative power in classification of the VMHC values in the HF and LTC may provide novel evidence to detect possible neuroimaging biomarkers in children with GTCS epilepsy syndrome.

### Disrupted Interhemispheric Functional Connectivity of the Default-Mode Network in Children With Generalized Tonic-Clonic Seizures

As neuroimaging techniques improve, modeling brain connectivity has received much attention and interest. The focus is on the brain organization and how alterations in connectivity underlie neurological disorder. In the past few years, investigating the functional interactions between regions has become a powerful tool to explore the human brain. In a resting state fMRI study, the disruption of FC networks in GTCS has been reported (Wang et al., 2011, 2019b; Liu et al., 2017; Parsons et al., 2020). Connectivity changes in a number of brain regions involved the HP, thalamus, insula, temporal cortex, cerebellum, precuneus, and medial prefrontal cortex. It is worth noting that the disrupted functional network connectivity in patients with GTCS was mainly related to the DMN (Song et al., 2011). The DMN is the most important brain network that is engaged in the modulation of internal mentation and external cognitive processes (Raichle et al., 2001). Previous studies have shown the deactivation and FC disruption of the DMN in patients with generalized seizures. For example, a previous study in idiopathic generalized epilepsy found enhanced negative FC in the precuneus of the thalamocortical pathway (Gong et al., 2021). The interaction between the thalamocortical pathway and the DMN was disrupted in the generalized epilepsy. A study using individual component analysis found both decreased and increased FC in the DMN and dorsal attention network in patients with GTCS (Wang et al., 2011). Additionally, a previous study found that DMN connectivity is altered in both generalized and focal epilepsies (Yang et al., 2021). Functional abnormalities in the DMN area are common characteristic among epilepsy syndromes. Understanding the brain connectivity of the DMN in epilepsy is particularly important, given that seizures may disrupt the brain network. In the present study, we used the VMHC method to detect interhemispheric connectivity changes in children with GTCS. The children with GTCS showed a significant decrease in VMHC in the superior occipital gyrus, HP, fusiform, putamen, amygdala, angular, LTC, middle temporal gyrus, and inferior and middle frontal gyrus. Because most of these regions are part of the DMN, this reduced interhemispheric connectivity in the DMN may be related to the impaired cognitive abilities that characterize children with GTCS. One of our recent studies using whole brain connectivity has found that the functional connectivity between bilateral middle temporal gyrus showed a significant decrease in children with GTCS (Li et al., 2020a). In the present study, the aberrant interhemispheric functional connectivity results in the DMN of children with GTCS were consistent with these previous functional interaction studies in GTCS. This result indicates that functional reorganization within the DMN rise may give rise to GTCS in children with epilepsy. The brain connectivity results were also supported by previous neuroimaging studies on brain activity of GTCS. A simultaneous EEG-fMRI study found that the patients with GTCS showed negative activation in the posterior cingulate gyrus, precuneus, and lateral parietal cortex and incomplete synchronized activities in the medial frontal cortex when the generalized spike wave appeared (Hamandi et al., 2006). Interictal epilepsy activity may reduce functional integration in the DMN, as shown in the present study. Another study using the regional homogeneity method found that the patients with GTCS showed regional homogeneity changes bilaterally and symmetrically in the precuneus, posterior cingulate gyrus, anterior cingulate gyrus, inferior parietal lobe, inferior frontal gyrus, and putamen (Zhong et al., 2011). One of our recent studies has also found that children with GTCS showed a significant decrease in spontaneous brain activity in the bilateral angular and left inferior and middle temporal gyrus (Wang et al., 2018). Many of these affected brain regions are components of the DMN. The long-term injurious effects of epileptic action may induce decreased regional synchronization and spontaneous brain activity in the DMN of patients with GTCS. This explanation can also be used in the present study to explain the significant decrease in interhemispheric connectivity in the DMN of children with GTCS.

In the present study, the VMHC results were not in accord with the two previous studies in adults with a similar method. In these two previous studies, adults with GTCS showed significant enhancement of the VMHC in the angular cortex, cuneus, prefrontal cortex, and anterior cingulate cortex (Ji et al., 2014; Yang et al., 2014). A significant decrease in the VMHC was found in adults with GTCS in the olfactory, supramarginal, inferior frontal gyrus, and temporal lobe in one study but not in the other. There were multiple changes in VMHC in the DMN in these previous studies. In this study, we only detected a significant decrease in interhemispheric connectivity in children with GTCS, especially in the DMN. For patients with GTCS, the interhemispheric connectivity changes of the DMN in the present study were different from these two previous studies. One possible reason for the above inconsistency may be that the research subjects were different. While previous studies focused on adults with GTCS, this study focused on children with GTCS. Childhood is a specific period with fast development of the brain. Chronic epilepsy can cause functional reorganization. A previous study indicated that the default regions in human brain integrate into a cohesive, interconnected network over development (Fair et al., 2008). The functional integration of the DMN is weak at a child’s age, and then is stronger over development. Chronic epilepsy may disturb the normal development of the DMN in children with GTCS. Thus, the FC between the bilateral regions of the DMN showed a decrease in the children with GTCS in this study. For adults with GTCS, the weak connectivity of the DMN gradually strengthened over development. Additionally, the seizure onset ages and the epilepsy durations were different among these studies. In the present study, the mean seizure onset age was approximately 3 years, and the mean epilepsy duration was approximately 2.5 years. In these two previous studies with VMHC methods, the mean seizure onset ages were approximately 13–17 years, and the mean epilepsy durations were approximately 3–7 years. A previous study showed that early and later ages of the seizure onset have differential impacts on brain resting-state organization (Doucet et al., 2015). The adult patients in the above previous studies had a longer epilepsy duration than the patients in the present study, which may indicate that adult patients have a long period to restore the function of the DMN. In the present study, the ages of the seizure onset were small, and the epilepsy durations were relatively short in children with GTCS. The seizure onset ages and the epilepsy durations may be another factor that induced the significant decrease in VMHC in the DMN in the present study.

A previous study has shown that motion-associated differences in brain connectivity cannot fully be attributed to motion artifacts but, rather, also reflect individual variability in functional organization (Zeng et al., 2014). The correlate of head motion consists of reduced distant functional connectivity primarily in the default network areas in individuals with high head motion. In the present study, we did not find a significant difference in head motion FD values between the two groups. According to the common suggestions, we also used head motion parameters as covariates during the analysis. This step can eliminate the effect of head motion on brain connectivity as much as possible. As the sample size of the present study is relatively small, the motion-associated differences in brain connectivity between the two groups are not shown. Future studies should consider this question with larger sample sizes.

### Altered Topological Organization of the Default-Mode Network in Children With Generalized Tonic-Clonic Seizures

In the present study, the graph theory method was used to map the topological properties of the DMN. Our results showed that both children with GTCS and TDC have a small-world topology in the functional DMN. Such a small-world topology in the whole brain network has been related to normal human cognitive functioning (He and Evans, 2010) and other pathological states (Fornito et al., 2015; Li et al., 2020a). Previous neuroimaging studies on epilepsy also found that patients with idiopathic generalized epilepsy or GTCS demonstrated a small-world property of the functional and structural networks (Zhang et al., 2011; Liao et al., 2013). Therefore, the graph results in this study indicated that the functional networks of the DMN in both groups showed high efficiency in information processing and transfer. Notably, we were only interested in the DMN but not the whole brain network in this study. Compared with the TDC, children with GTCS showed a reduction in Eg and an increase in Lp in the DMN. Group differences in regional topological organization were also found in the areas of the bilateral TPJ, bilateral LTC, left TempP, and left HF. The change trend of topological organization was consistent with a previous study that focused on the integration of the DMN in adults with GTCS (Song et al., 2011). This previous study showed that the degree and FC of brain areas within the DMN were significantly reduced in patients with GTCS. One study across the generalized types found that the DMN showed more significantly altered connectivity than other resting state networks in patients with idiopathic generalized epilepsies (Parsons et al., 2020). The disruption of functional network organization in the DMN is a common feature in generalized epilepsy. This evidence from previous neuroimaging studies, together with the present graph results, suggests that children with GTCS may have a less optimized network organization of the functional DMN than the TDC.

We also used the NBS approach for the functional connection of the DMN to localize the specific connected components. Importantly, the NBS graph connectivity analysis was consistent with the VMHC results in identifying that the interhemispheric connectivity between bilateral ROIs (left HF vs. right HF and left LTC vs. right LTC) was decreased significantly in patients. Our findings are in good agreement with previous reports that showed a significant decrease in interhemispheric FC in the DMN of adults with GTCS (Song et al., 2011; Ji et al., 2014; Li et al., 2016). In addition to the connectivity results from adults with GTCS supporting our results, recent graph theory results in children with GTCS have also supported the present graph connectivity results. One recent study using graph theory analysis on the GM structural covariance network has found that children with GTCS showed significant alterations in nodal betweenness in the right thalamus, bilateral temporal pole, and some regions of the DMN (Li et al., 2020b). Another recent graph theory study on the functional network has found a significant decrease in *C*_p_ and nodal local efficiency in the bilateral putamen, MTG, and temporal pole of the MTG of children with GTCS (Li et al., 2020a). Bilateral changes in nodal characteristics and the interhemispheric connectivity of the regions belonging to the DMN were the common results among these graph theory studies in children with GTCS. The interpretation for the disrupted interhemispheric connectivity in DMN may be that the capacity of brain communication between the bilateral regions of DMN was reduced. Taken together, our graph theory and connectivity findings in DMN tend to imply that GTCS in children is characterized by reduced interhemispheric connectivity within the DMN. The importance of the interhemispheric connection results is discussed in the following section by combining the correlation and machine learning results.

Here, we should notice that the present work is an extension of our previous research (Li et al., 2020a). The sample size was enlarged, and the research objectives were also different. The previous study is mainly focused on the whole-brain topological organization, while the present study is focused on the functional homotopy and topological properties within the DMN. And, also, the graph results of the present study can further enrich our understanding the disrupted interhemispheric connectivity in DMN.

### Clinical Relevance of Functional Homotopy in the Default-Mode Network in Children With Generalized Tonic-Clonic Seizures

In the present study, it is worth noting that the average VMHC values in the bilateral HF and LTC were significantly correlated with the epilepsy duration. Previous studies have confirmed that epilepsy duration is one of the main clinical factors affecting a patient’s functional organization (Wang et al., 2011, 2018, 2019b; Liu et al., 2017). In these previous studies, the FC between the regions within the DMN or between the DMN and other subnetworks of the brain showed negative correlations with the epilepsy duration. In adults with GTCS, regional synchronization and functional rich-club connectivity in the regions of the DMN were also found to be negatively correlated with the epilepsy duration (Zhong et al., 2011; Li et al., 2016). The correlation result in the present study was consistent with these previous adult studies and indicated that the functional reorganization in the DMN can reflect the effect of epilepsy duration in both adults and children with GTCS. Children with longer illness durations showed lower VMHC between the bilateral HF and LTC. The VMHC-duration correlation results in the present study further support the proposal that the damaging effect of GTCS might disrupt the functional homotopy of the DMN in children (Li et al., 2020a,b). The significant negative correlation between the epilepsy duration and VMHC values in the bilateral HF and LTC further confirmed that reduced interhemispheric connectivity in DMN can reflect the seizure effect on the information communication in children with GTCS.

In addition to the epilepsy duration, the onset age of the first seizure is another factor that affects the resting-state connectivity of patients with epilepsy (Doucet et al., 2015). This previous study has shown that the age of the seizure onset can lead to different perturbations of network modularity and connectivity at the global and local levels in temporal lobe epilepsy, with different implications for regional plasticity and adaptive reorganization. A recent study has found that the FC between the insular and thalamic projections was significantly correlated with the onset of illness in patients with GTCS (Gong et al., 2021). The onset age of illness can affect the brain network organization in patients with GTCS. In the present study, we further found that the average VMHC values in bilateral HF and LTC were positively correlated with the onset age of first seizure in children with GTCS. In line with these previous correlation findings, these partial correlation results indicated that children with later onset ages would show less disruption of interhemispheric connections in the DMN. Additionally, the children with a later onset age would have less cognitive impairment. Thus, both correlation results in the present study imply that the dysfunction of the interhemispheric connectivity in the DMN would play an important role in the understanding of the neuromechanism of functional disorder in GTCS.

In the present study, VMHC values in bilateral HF and LTC also showed a positive correlation with the FC values between the bilateral HF and LTC. VMHC is a new validated neuroimaging index that can quantify functional homotopy by providing a voxel-wise measure of connectivity between hemispheres (Zuo et al., 2010). The brain’s essential functional architecture can be measured by this index. Resting-state FC is measured with the correlations in MRI signals to examine the synchronizations between spontaneous neurophysiological events in spatially remote brain regions (Biswal et al., 1995). Thus, the positive correlation between the VMHC and FC of the bilateral HF and LTC can be easy to understand. However, significant partial neuroimaging-behavioral correlations were detected for the VMHC but not for the FC in the bilateral HF and LTC. This result can be explained by the different analysis processes of these neuroimaging indices. The VMHC quantifies the resting-state FC between each voxel in one hemisphere and its mirrored counterpart in the other. A voxel-wise measure of the VMHC values was calculated and averaged to obtain the mean VMHC between the bilateral HF and LTC. For the average FC, the average MRI signals were extracted from the predefined ROIs of the HF and LTC. The FCs between the bilateral HF and bilateral LTC were calculated and averaged to acquire final average FC. The VMHC used a voxel-wise measure that can reflect the signal fluctuation between different voxels in the same region. This index is more sensitive than the FC index in reflecting the interhemispheric organization in children with GTCS. This opinion should be confirmed in further studies.

### Clinical Relevance of Network Alterations in Children With Generalized Tonic-Clonic Seizures

To confirm the importance of the interhemispheric connection results, a machine learning method was used to detect whether the functional homotopy of bilateral HF and LTC can classify the children with GTCS from the TDC. Machine learning is an emerging trend in medicine that can be used to develop and/or optimize clinically useful algorithms for clinical medicine and basic research. In recent years, an increasing number of clinical and experimental applications of machine learning methods for epilepsy have become available in the diagnosis of epilepsy, surgical management of epilepsy, and medical management of epilepsy (Abbasi and Goldenholz, 2019; Sone and Beheshti, 2021). Machine learning techniques have enabled imaging analysis and epilepsy diagnosis from a wide range of clinical data. In addition to the clinical manifestations and EEG characteristics, the specificity of brain neuroimaging in epilepsy can also be used as a feature in epilepsy diagnosis. A common application of machine learning for brain imaging in epilepsy is the differentiation between brains with epilepsy and healthy brains (Bharath et al., 2019; Zhou et al., 2020). A previous study in our group using machine learning found that the GM volume and fALFF value in the right thalamus can be used as features to discriminate children with GTCS from the TDC (Wang et al., 2018). In the present study, the VMHC values in the bilateral HF and LTC were set as features of the machine model, and the SVM model was used to discriminate between the two groups. We classified the children with GTCS from the TDC by the linear SVM classifier, corresponding to an accuracy of 89.34% based on the VMHC values of bilateral HF and LTC. The machine learning results were consistent with these previous studies and demonstrated that specific changes in interhemispheric connectivity in the DMN allowed accurate discrimination between children with GTCS and the TDC at the level of an individual. Combining the significant correlation results, the VMHC values of bilateral HF and LTC can be considered as biomarkers to classify children with GTCS from TDC at the individual subject level. The disruption of interhemispheric connectivity in the DMN has potential value in the application of epilepsy diagnosis.

### Limitations

Several limitations of this study should be noted. First, all children with GTCS were medicated in the present study. A previous study detected that using antiepileptic drugs can affect normal neuronal function and produce cognitive impairment in children (Ijff and Aldenkamp, 2013). The present results may also be affected by antiepileptic drugs. Future studies should be conducted to exclude the effect of antiepileptic drugs on interhemispheric connectivity in children with GTCS. Second, 18 children under the age of 4 were sedated with 10% chloral hydrate during image collection. Although this approach can improve the success rate of neuroimaging data collection in children, the use of sedatives in younger children may also affect brain connectivity. Future studies need to be conducted to clarify the effect of sedative drugs on brain connectivity. Third, the sample size of the patient group was relatively small compared with the size of the TDC. During the data connection, some patients were unwilling to cooperate with the MRI scan, and we could not obtain valid data from these subjects. Future studies should collect larger sample sizes to provide further insights into the issue. Finally, it should be noted that all analyses in the present study were performed on the same dataset. Although the functional homotopy of bilateral HF and LTC has the high ability to classify the children with GTCS from the TDC by the linear SVM classifier, the machine learning results were not validated by a new sample of testing data. Future studies should collect more samples as testing data to verify the stability of the present classification model.

## Conclusion

In the present study, we combined the VMHC method and graph theory to investigate the interhemispheric organization of the DMN in children with GTCS. Although both the children with GTCS and the TDC exhibited small-world topology in the functional network of the DMN, the children with GTCS showed changes of the optical topological organization in the DMN. Both VMHC and graph connectivity analyses found that the children with GTCS showed a significant decrease in interhemispheric connectivity in the bilateral HF and LTC. The brain connectivity results indicate that the brain functional homotopy, network architecture, and communities in the DMN of the children with GTCS were changed significantly. Importantly, we found that the functional homotopy of bilateral HF and LTC was significantly correlated with the epilepsy duration and the age of the seizure onset. This neuroimaging-behavioral coupling may reflect seizure effects on brain organization in children with GTCS. VMHC values in the bilateral HF and bilateral LTC exhibited discriminative power in the classification of children with GTCS from the TDC. Machine learning results reflect that decreased VMHC in the DMN can be considered as a biomarker to classify children with GTCS from the TDC at the individual subject level. In conclusion, dysfunction of the interhemispheric connectivity in the DMN will play an important role in the understanding of the neuromechanism of GTCS in children and has potential value in the diagnosis of epilepsy.

## Data Availability Statement

The original contributions presented in the study are included in the article/Supplementary Material, further inquiries can be directed to the corresponding authors.

## Ethics Statement

The studies involving human participants were reviewed and approved by the Ethical Committee of the Shenzhen Children’s Hospital. Written informed consent to participate in this study was provided by the participants’ legal guardian/next of kin.

## Author Contributions

YL and QC conceived and designed the experiments. QC performed the experiments. YL analyzed image data, sorted the results, and wrote and reviewed the manuscript. QC, BQ, and JC were responsible for patient management and conceptualized the study. All authors contributed to the article and approved the submitted version.

## Conflict of Interest

The authors declare that the research was conducted in the absence of any commercial or financial relationships that could be construed as a potential conflict of interest.

## Publisher’s Note

All claims expressed in this article are solely those of the authors and do not necessarily represent those of their affiliated organizations, or those of the publisher, the editors and the reviewers. Any product that may be evaluated in this article, or claim that may be made by its manufacturer, is not guaranteed or endorsed by the publisher.

## Footnotes

1
http://www.nitrc.org/projects/gretna/

2
http://www.mlnl.cs.ucl.ac.uk/pronto
