# Supplementary material for: Impaired Functional Homotopy and Topological Properties Within the Default Mode Network of Children With Generalized Tonic-Clonic Seizures: A Resting-State fMRI Study
Source: Front Neurosci. 2022 Jun 2;16:833837. doi: 10.3389/fnins.2022.833837 (PMC9201640; doi:10.3389/fnins.2022.833837)
Supplement: Supplementary file 1 [file Data_Sheet_1.DOCX]

**Supplement Table 1: Regions of Interest (17 DMN regions)**

| Regions | Abbreviation | x | y | z |
| --- | --- | --- | --- | --- |
| **PCC-aMPFC Core** | | | | |
| Anterior medial prefrontal cortex | aMPFC | -6 | 52 | -2 |
| Posterior cingulate cortex | PCC | -8 | -56 | 26 |
| **dMPFC** **Subsystem** | | | | |
| Dorsal medial prefrontal cortex | dMPFC | 0 | 52 | 26 |
| Temporal parietal junction | TPJ | ±54 | -54 | 28 |
| Lateral temporal cortex | LTC | ±60 | -24 | -18 |
| Temporal pole | TempP | ±50 | 14 | -40 |
| **MTL Subsystem** | | | | |
| Ventral medial prefrontal cortex | vMPFC | 0 | 26 | -18 |
| Posterior inferior parietal lobule | pIPL | ±44 | -74 | 32 |
| Retrosplenial cortex | Rsp | -14 | -52 | 8 |
| Parahippocampal cortex | PHC | ±28 | -40 | -12 |
| Hippocampal formation | HF | ±22 | -20 | -26 |

Note: Coordinates are based on the Montreal Neurological Institute coordinate system. Because regions are 8 mm spheres, Brodmann areas list approximate locations for reference.
